# Cryo-Electron Tomography of Marburg Virus Particles and Their Morphogenesis within Infected Cells

**DOI:** 10.1371/journal.pbio.1001196

**Published:** 2011-11-15

**Authors:** Tanmay A. M. Bharat, James D. Riches, Larissa Kolesnikova, Sonja Welsch, Verena Krähling, Norman Davey, Marie-Laure Parsy, Stephan Becker, John A. G. Briggs

**Affiliations:** 1 Structural and Computational Biology Unit, European Molecular Biology Laboratory, Heidelberg, Germany; 2 Institut für Virologie, Philipps-Universität Marburg, Marburg, Germany; Institut Pasteur, France

## Abstract

Several major human pathogens, including the filoviruses, paramyxoviruses, and rhabdoviruses, package their single-stranded RNA genomes within helical nucleocapsids, which bud through the plasma membrane of the infected cell to release enveloped virions. The virions are often heterogeneous in shape, which makes it difficult to study their structure and assembly mechanisms. We have applied cryo-electron tomography and sub-tomogram averaging methods to derive structures of Marburg virus, a highly pathogenic filovirus, both after release and during assembly within infected cells. The data demonstrate the potential of cryo-electron tomography methods to derive detailed structural information for intermediate steps in biological pathways within intact cells. We describe the location and arrangement of the viral proteins within the virion. We show that the N-terminal domain of the nucleoprotein contains the minimal assembly determinants for a helical nucleocapsid with variable number of proteins per turn. Lobes protruding from alternate interfaces between each nucleoprotein are formed by the C-terminal domain of the nucleoprotein, together with viral proteins VP24 and VP35. Each nucleoprotein packages six RNA bases. The nucleocapsid interacts in an unusual, flexible “Velcro-like” manner with the viral matrix protein VP40. Determination of the structures of assembly intermediates showed that the nucleocapsid has a defined orientation during transport and budding. Together the data show striking architectural homology between the nucleocapsid helix of rhabdoviruses and filoviruses, but unexpected, fundamental differences in the mechanisms by which the nucleocapsids are then assembled together with matrix proteins and initiate membrane envelopment to release infectious virions, suggesting that the viruses have evolved different solutions to these conserved assembly steps.

## Abstract 2

Ultrastructural analysis of a filovirus assembling within infected eukaryotic cells reveals differences in structure and assembly mechanisms between related RNA viruses.

## Author Summary

Marburg virus and Ebola virus cause severe hemorrhagic fever in humans and non human primates. They are members of the family of Filoviridae, and part of the order *Mononegavirales*, which includes other important human pathogens: rabies virus, respiratory syncytial virus, and measles virus. All of these viruses contain a single-stranded RNA genome enclosed within a helical protein assembly called the nucleocapsid. In this study, we used electron microscopy to image individual Marburg virus particles, allowing us to determine the architecture of the nucleocapsid and the positions of the viral proteins. We found that the nucleocapsid structure contained a “barbed” and a “pointed” end, and was similar to that of the related family of bullet-shaped Rhabdoviridae, which includes rabies virus. When we generated 3-D images of virus particles frozen in the act of assembling and budding from infected cells, however, we found that despite their structural similarities, Marburg virus particles are released from infected cells with the pointed end of the nucleocapsid facing out, whereas rabies virus is released with its barbed end facing out, suggesting different budding mechanisms. This study illustrates not only how electron microscopy of frozen samples can resolve structural information in vivo, but it provides knowledge of the structure of a filovirus and its assembly pathway, generated by direct 3-D imaging of infected cells, revealing similarities and differences in the assembly and budding mechanisms of members of *Mononegavirales*.

## Introduction

Members of the virus order *Mononegavirales* represent a critical challenge to human health. The order contains major human pathogens including paramyxoviruses such as measles virus (MeV), mumps virus, and respiratory syncytial virus (RSV); rhabdoviruses such as rabies virus (RABV); and filoviruses such as Ebola virus (EBOV) and Marburg virus (MARV) [1]. Members of the order are characterized by a single-stranded, negative-sense, non-segmented RNA genome, which is replicated from a nucleoprotein (NP)-RNA complex by the viral L protein, an RNA-dependent-RNA-polymerase, and a polymerase cofactor (the phosphoprotein P in rhabdoviruses and paramyxoviruses) [2]. The newly synthesized RNA is bound by NP and assembles together with other viral proteins to form a helical nucleocapsid (NC). The NC, matrix protein, and other components assemble at and bud through the plasma membrane of an infected cell to form virions enveloped by a host-cell-derived membrane.

The core region of *Mononegavirales* NPs is made up of two primarily alpha-helical domains, with the RNA bound at the interface between the two domains [3]. The NPs assemble into helical NCs where the two domains of each NP monomer protrude at an angle to the helical axis to give the NCs a strong structural polarity with characteristic “pointed” and “barbed” ends by analogy with actin [4]–[6]. These consistent features are balanced by substantial differences. The diameters of the NCs vary among the *Mononegavirales*, indicating different numbers of NP monomers per turn of the helix; there can even be variability in the number of NPs per turn within a single virion [7]–[9]. The number of RNA bases bound per NP also varies, from six or seven in the paramyxoviruses to nine in the rhabdoviruses, reflecting differences in genome replication mechanisms. Most strikingly, the NCs are enveloped and released into virions, which show major differences in structure and morphology. Rhabdoviruses like vesicular stomatitis virus (VSV) and RABV form bullet-shaped particles with a defined diameter and length [10]. Other families in the order form more heterogeneous particles. Paramyxoviruses like MeV, Sendai virus, (SeV) and RSV form mostly round-shaped particles of variable size, but filamentous forms may also be observed [11],[12]. Filoviruses adopt a range of morphologies from filamentous, through “six-shaped” to round particles [13],[14].

Only for one member of the order, the prototypical rhabdovirus VSV, is a detailed structural description of the virion available. VSV assembles regularly shaped particles, and this regularity permits application of single-particle cryo-electron microscopy (cryoEM) analysis, a method that cannot be applied to heterogeneous virions. The resulting 3-D structure shows that the helical NC makes direct, regular interactions with matrix, potentially allowing the NC helix to define the shape of the virion through direct contact [5]. The NC has a bullet-shape, with a dome-like tip. Interaction of the M protein with the dome-like NC tip has been suggested to mediate envelopment of viral NC to form a protruding, bullet-shaped particle that is then pinched off [15]. It is tempting to speculate that this may be a conserved mechanism also adopted by other members of *Mononegavirales* including the filoviruses. It has been shown that the filovirus NC associates laterally with the membrane, before envelopment initiates from an end of the NC, leading to a perpendicularly protruding intermediate that pinches off from the infected cell [14],[16]. It is not known whether one end of the NC specifically initiates envelopment or whether it is initiated randomly from either end. It is also not clear if the filovirus NC has a structure comparable to the dome-like tip of the VSV NC. To understand the extent to which the knowledge obtained from the VSV structure can be applied to other *Mononegavirales* members requires that structural information is obtained for other, more heterogeneously shaped families within the order. Here we have addressed this need by describing the structure and assembly of MARV, a filovirus.

The family *Filoviridae* contains two genera: MARV and EBOV. Both viruses are highly pathogenic, causing hemorrhagic fever in infected humans, with high mortality rates. They are classified as highest priority bioterrorism agents by the Centers for Disease Control and Prevention (USA). Filovirus research is complicated by the need to perform experiments under biosafety level 4 (BSL-4) conditions. Nevertheless, biochemical, structural, and functional studies have explored the properties of the viral proteins and their role in assembly and budding [17]. As a biological system, a filovirus is remarkably simple. The 19 kb RNA genome and the viral proteins NP, VP30, VP35 (the polymerase cofactor), VP24, VP40 (the matrix protein), and L are enveloped by a lipid membrane containing trimers of the glycoprotein (GP) [18]. VP30, VP35, and L have been assigned as components of the NC based upon salt dissociation and biochemical analysis of isolated MARV, and on the same basis VP24 and VP40 were described as the minor and major matrix proteins, respectively [19]–[21]. For EBOV, it has been shown that NP, VP24, and VP35 are necessary and sufficient to form NC-like helical structures [22],[23]. EBOV VP40 and MARV VP40 can assemble and bud enveloped filamentous virus-like particles (VLPs) in the absence of other viral proteins [21],[24]. VP40 VLPs have been reported to be slightly narrower in the absence of a NC, suggesting that an interaction is present between the two [25]. More precise information about the location and arrangement of the proteins within virions is not available. No crystal structure of the NP or low-resolution structure of the NC of filoviruses is available. The structural relationship between the matrix protein VP40 and NP in filoviruses, and potential importance of this relationship in defining virion structure, therefore remains undescribed.

A structural understanding of filoviruses has previously been complicated by the need to prepare viruses under BSL-4 containment conditions, and by difficulties inherent in applying the methods for 3-D structural studies to heterogeneous virions. Single-particle cryoEM methods used to derive the structure of VSV cannot be applied to the heterogeneous particles of MARV. Here we have applied multiple electron microscopy (EM) approaches including cryoEM, cryo-electron tomography (cryoET) combined with sub-tomogram averaging, and immuno-electron microscopy (IEM) to describe the structure of purified MARV in 3-D. The data permit determination of the location and arrangement of viral proteins within MARV, the architecture of the NC, minimal assembly determinant, RNA packaging stoichiometry, and a flexible relationship between the NC and viral matrix protein. This study provides the first detailed 3-D description of the structure of a BSL-4 pathogen, revealing principles of structure and assembly, which are likely to extend to other heterogeneously shaped *Mononegavirales*. CryoET can also be used to study the structure of assembly components within cells [26]–[29]. We have therefore applied cryoET to also visualize MARV budding from the filopodia of infected cells in 3-D. By combining cryo-ET with sub-tomogram averaging we were able to resolve the 3-D structure of the NC in situ, during membrane envelopment, showing that the NC has a defined orientation during transport and budding and implying a mechanism of virus assembly, which is fundamentally different than that in the rhabdoviruses.

## Results

### Virion Morphology and the Arrangement of Viral Proteins

**Figure 1:**
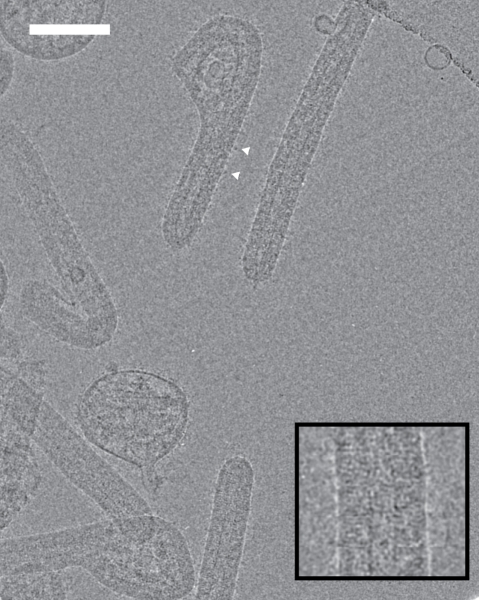
CryoEM of MARV. CryoEM image of the morphologies observed in MARV samples. Scale bar, 100 nm, electron density black. Arrowheads indicate examples of spike-like membrane protrusions. Inset: magnified view of part of a filamentous particle showing striations under the membrane.

MARV particles were harvested from the supernatant of infected Vero cells 3 d post-infection, purified by centrifugation, and fixed in paraformaldehyde (PFA) prior to release from the BSL-4 laboratory (Materials and Methods). The purified preparation was imaged using cryoEM. Three morphological forms of the virus were observed in the samples: filamentous (30%, *n* = 128), six-shaped (37%), and round particles (33%) (Figure 1). All of these had a membrane with spike-like protrusions approximately 10 nm in length. This is slightly longer than the size described for the non-glycosylated EBOV GP [30], since the MARV GP on virions should be fully glycosylated. The filamentous particles and the straight sections of the six-shaped viruses contained NCs along most of their length and had approximately hemispherical tips. In round particles, and in the curved sections of six-shaped virions, it was possible to detect short sections of NC close to the viral membrane, in agreement with previous studies of NC morphology within virions [14]. A striated density located between the NC and the viral membrane was observed in the filamentous and six-shaped particles. The filamentous particles had a mean length of 892 nm (SD 63 nm) and a mean diameter of 91 nm (SD 6 nm), slightly longer than earlier studies, since cryo-EM avoids the compression and shrinkage effects associated with sectioning and imaging plastic embedded samples [14],[31].

**Figure 2:**
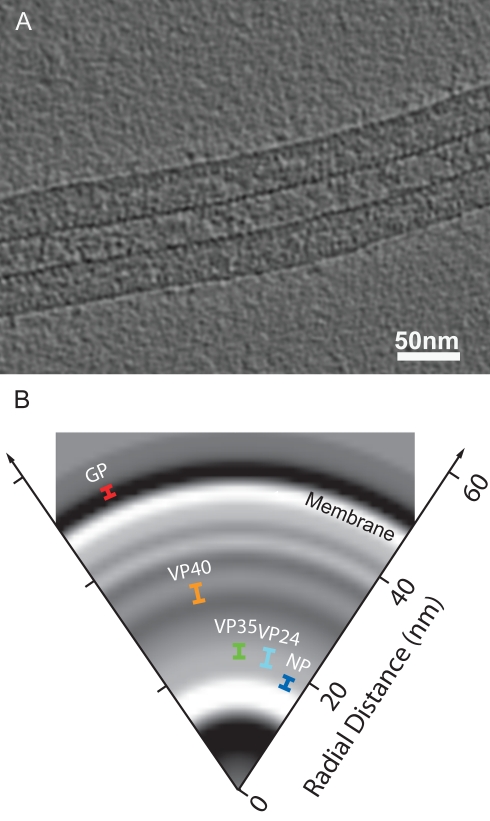
CryoET of MARV and mapping of viral proteins. (A) A slice through a tomogram showing a section of a MARV virion. Electron density is black. (B) The radial positions of the proteins in the virion, represented by the mean, Gaussian-corrected positions of the gold beads in IEM have been superimposed onto a radial density slice of the virions produced from the aligned subtomograms (Text S1). The length of the bar represents the error associated with each IEM measurement. Electron density is white. See Figure S1 and Table S1 for further details.

To describe in detail the structural arrangement of the viral proteins within the virion, we collected cryoET data on free virions and produced 3-D tomograms from them (Figure 2A). From these tomograms, subtomograms (or sub-volumes) were extracted along the length of filamentous particles and averaged to generate a radial density profile of the virion (Text S1), from which it was possible to discern distinct layers of density (Figure 2B). The innermost layer of density in the radial-density profile corresponds to the NC visible in our cryo-micrographs, and the outermost to the lipid membrane. As expected, there was no density layer corresponding to GP because GP is sparsely distributed on the surface of the particle rather than forming a continuous layer.

In order to assign the observed layers to individual proteins, we performed thin section IEM on the purified virus sample [32]. Sections were immunolabelled using antibodies against either VP24, VP35, VP40, GP, or NP followed by protein-A gold [33] and examined by EM. The distances of the gold beads from the centre of the radial cross-section were measured, and used to calculate the radial distribution of each of the epitopes (Figures S1 and 2B and Table S1). This experiment allowed us to assign the innermost density to NP, and the adjacent density band to VP24 and VP35 (Figure 2B). The VP40 epitope is positioned at the innermost of two layers of density immediately underneath the membrane. A comparison with GP-VP40 VLPs confirms that the outer of these two layers is also contributed by VP40 (see below). The GP signal is seen slightly outside the outer membrane, as expected.

### NC Structure

**Figure 3:**
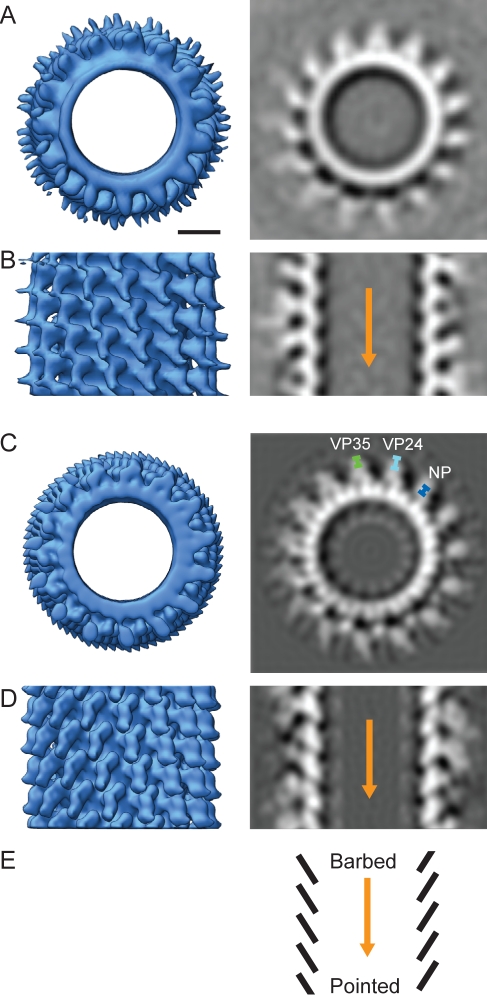
Reconstruction of the MARV NC. (A–B) Reconstruction of the MARV NC from cryoET and subtomogram averaging. (A) The NC helix is shown viewed along the helical axis as an isosurface (left) and as a section through the density (right). All isosurfaces are displayed at a contour level of 1.5 σ away from the mean. Scale bar, 10 nm. Electron density is white. (B) Side-on view of helix. The orange arrow is directed towards the pointed end of the NC helix. (C–D) Reconstruction of the MARV NC from 2-D helical reconstruction techniques in the same orientations as (A–B). The radial positions of the NC proteins as determined from the mean location of the gold beads earlier in Figure 2B have been superimposed (C, right). (E) Schematic representation of the NC highlighting the “pointed” and “barbed” end of the helix. See Figures S2 and S3.

After assignment of the NC density, we wanted to explore its structure. Established 2-D helical reconstruction techniques are not directly applicable to the NC helix within the virion, because the surrounding proteins and membrane confound the image and prevent determination of the helical parameters. Instead we applied novel reference-free subtomogram averaging approaches to generate 3-D reconstructions of NCs from within 30 individual virions (Materials and Methods). These reconstructions showed that the NC has an inner density forming a strong and continuous left-handed, single-start helix inclined to the filament axis and roughly “boomerang”-shaped protrusions extending outwards from this inner density. The pitch of the NC helix was 7.5 nm±0.2 nm. Twenty-one of the analyzed NCs had a symmetry of 14.8 protrusions per turn, seven NCs had 15.8 protrusions per turn, and two NCs had 13.8 protrusions per turn. This indicated that the individual subunits of the helical NC have sufficient flexibility to adopt slightly different overall symmetries. One case was observed in which there was a change in the symmetry from 14.8 to 15.8 protrusions per turn within a virion (Figure S2). The NCs with 15.8 protrusions per turn were combined into a single 3-D reconstruction with a resolution of 4 nm (unpublished data); those with 14.8 protrusions per turn were combined into a single 3-D reconstruction with a resolution of 3.4 nm (Figure 3A–B). Both reconstructions show the same features.

The subtomogram averaging analysis therefore allowed us to determine the pitch of the NC helix and the number of subunits per turn. We could then use this information to apply helical reconstruction techniques to derive a higher resolution 3-D structure of the NC from 2-D micrographs of purified virions [34]. We sorted the 2-D data according to symmetry (Text S1) and obtained 3-D reconstructions for the different symmetries of the NC. The reconstruction of the predominant 14.8 protrusion per turn symmetry had 2.5 nm resolution (Figure 3C–D). The resolution of the reconstruction is highest at a radius coincident with the inner layer of the NC, and falls off gradually outwards towards the membrane (Figure S3), indicating that the innermost NC layer is thus the most rigidly ordered part of the structure, and variability and flexibility increases with increasing distance from the centre of the helix. At this resolution, the innermost helical layer of the NC is resolved into 30 separate lobes of density per turn (compare Figure 3C right with 3A right), with two lobes of density per boomerang-shaped protrusion. The protrusions emanate from each alternate interface between the inner lobes. The inclination of the lobes of density in the inner helix relative to the helical axis defines the “pointed end” and the “barbed end” of the NC (Figure 3B, 3D, and 3E), as seen in the NCs of other *Mononegavirales*. Comparison of the 3-D reconstructions with our IEM data (Figure 2B) suggests that the protrusions correspond to the location of VP24 and VP35, and that the innermost density of the MARV NC reconstruction corresponds to NP.

**Figure 4:**
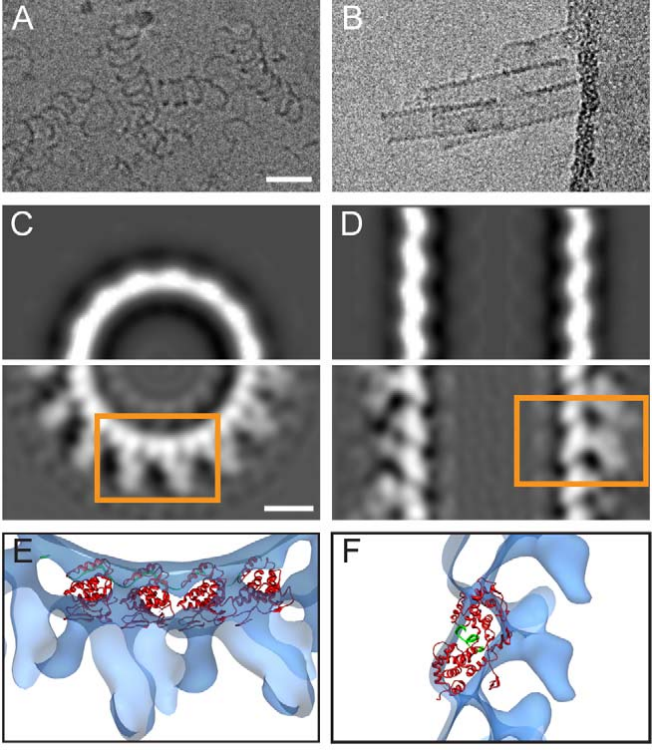
Assignment of the core region of MARV NP and fitting of VSV NP into the MARV NC density. (A) CryoEM image of MARV NP purified from 293T cells. Scale bar, 50 nm, electron density black. (B) CryoEM image of MARV NP(1–390) produced in a parallel experiment. (C–D) Comparison of the MARV NC reconstruction in Figure 2 with that of the MARV NP(1–390) helix. (C) A section perpendicular to the helical axis for the NC helix (lower panel) compared to the corresponding part from the NPΔ390 helix (upper panel). Scale bar, 10 nm, electron density white. (D) Corresponding section along the helical axis. (E–F) Fitting of the pseudo-atomic model of the VSV NP (PDB 2WYY with protein in red and RNA in green) into the full MARV NC reconstruction, after assignment of the NP density. The views shown correspond to the regions within the orange boxes in (C–D).

To confirm the assignment of the NP density, we imaged purified, recombinant MARV NP by cryoEM (Figure 4A). The oligomeric NP appears as loose coils with an approximate diameter of 30 nm as seen previously [35]. Based on bioinformatics analysis of the NP sequence (Figure S4), we expressed a truncated form of the MARV NP, containing only the core-conserved 390 N-terminal residues, which formed short rigid helices in dense clusters (Figure 4B). CryoET and subtomogram averaging indicated that these helices had the same diameter, hand, and pitch as the innermost density of the viral NC reconstruction, with a closely matching structure (Figure 4C–D), demonstrating that this layer is formed by the core-conserved N-terminal 390 residues of NP. Furthermore, it demonstrates that the core region of NP can define the helical parameters of the viral NC and assemble in the absence of any other viral proteins.

We hypothesized that each lobe in the inner layer corresponded to one copy of NP. No crystal structure of MARV NP is available, but the core-conserved region is expected to have structural homology to other *Mononegavirales* NPs. We therefore performed rigid-body fitting of four adjacent monomers, extracted together from the pseudo-atomic structure of the VSV NP helix (PDB ID 2WYY) [5],[36] into the innermost layer of the full MARV NC density we had reconstructed from within virions (Figure 4E–F and Movie S1, Text S1). The curvature of the NC helix and the characteristic inclination of the NP from the helix axis constrain the structure so that it can only be fitted as a rigid body in one position. The inclination of VSV NP from the helix axis matches the inclination seen in the 3-D reconstructions of MARV NC. One VSV NP fits into each lobe in the inner helix. High-resolution features such as the position of the RNA may differ between MARV and VSV, but the fit suggests striking architectural homology between the two NCs.

Together, the above observations allow us to calculate the number of RNA bases bound per NP. The average symmetry of the MARV NC helix is 14.96 protrusions per turn; therefore, it has on average 29.92 copies of the NP per turn. A virion of mean length 892±63 nm contains an NC of approximately 804 nm in length. The helix has a pitch of 7.52±0.19 nm per turn, meaning that an NC of mean length has approximately 107 turns, corresponding to approximately 3,170 copies of NP. Since the genome has a length of 19 kb, each NP monomer packages 6.0±0.2 RNA bases.

### Directionality of the MARV NC

**Figure 5:**
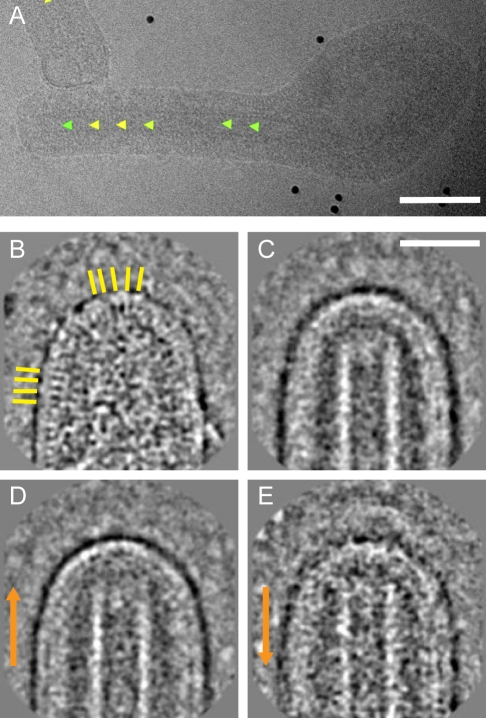
Directionality of the NC in free virions and structure of MARV tips. (A) A six-shaped MARV with arrowheads indicating the “pointed end” orientation assigned during helical reconstruction. Color represents cross-correlation coefficient (green∶high, yellow∶low) between extracted boxes and corresponding reprojections of the NC reconstruction. Scale bar, 100 nm, electron density black. (B) A representative extracted “pointed” MARV tip. Yellow lines highlight VP40 striations. Scale bar, 50 nm, electron density white. (C) Two-dimensional average of all extracted MARV tips. (D) Average of all “pointed” MARV tips. (E) Average of all “barbed” MARV tips. Orange arrows are directed towards the pointed end of the NC.

During the 2-D helical reconstruction of the NC, an orientation is assigned to each NC segment within each image. We mapped this directionality information back onto the original electron micrographs (Figure 5A). Out of 40 six-shaped virions where the image quality was appropriate for direction assignment, 33 had the “pointed” end of the NC towards the tip (the top) of the six and seven had the “barbed” end towards the tip of the six, indicating a preference of the pointed end of the NC to be towards the tip of the “six.”

Seven virions were identified that were more than twice the length of the average MARV particle, and therefore probably contained more than one RNA genome inside. In these virions, the NC did not change direction throughout their length (Figure S5), implying that both genomes were packaged with the same directionality.

### The Structure of the Virion Tip

To obtain a clearer view of the virion tips, 248 tips were computationally extracted from micrographs. An image of a virion tip is shown in Figure 5B. Striations are seen on the underside of the viral membrane, also in some cases in the curved part of the membrane at the tip. These striations are also seen in the GP-VP40 VLPs and correspond to VP40 [37]. All the extracted tips were then aligned and averaged with each other. The average of all viral tips is shown in Figure 5C. Based on our assignment of direction, we then averaged the pointed and barbed tips separately. These averages are shown in Figure 5D and 5E, respectively. The averages show that in neither case does the NC extend to the viral membrane, but rather ends approximately 44 nm before the inner side of the membrane. No dome structure capping the NC, equivalent to that described for VSV, was seen. The barbed tips were found to be less regular than the pointed tips (Figure S5B–D).

### The Arrangement of VP40

VP40 was not resolved in the NC reconstructions, indicating that, in contrast to the situation in VSV [5], the matrix layer does not follow the helical symmetry of the NC in MARV. A reconstruction of the VP40 lattice could not be generated by reference-free subtomogram averaging methods, likely due to variability and flexibility in the lattice. Instead, we applied 3-D Fourier analysis methods to measure local regularity within the VP40 lattice (Text S1). These methods revealed the presence of regularly spaced features within both inner and outer VP40 layers (Figure S6).

The expression of only VP40 and GP in cells leads to the assembly and release of filamentous membrane-bound VLPs [38]. We purified GP-VP40 VLPs and subjected them to the same cryo-ET and Fourier analysis. The particles were narrower (71 nm, SD 7 nm) than the virions. They also exhibited inner and outer layers of VP40 density with regularly spaced features generally similar to that in the virion but showing some clear differences (Text S1). Most strikingly, only within the virion does the VP40 layer contain features that repeat regularly around the circumference of the virion. These observations indicate a significant change in VP40 conformation occurs in the presence of the NC.

### CryoET of NC Assembly and Budding in Infected Cells

Having built up an in-depth picture of the structure of the NC in the free virion, we wanted to investigate the structural changes occurring during membrane association and envelopment. Infected HUH-7 cells (Materials and Methods) were fixed, vitrified by plunge-freezing, and examined using cryo-ET. The cells appeared to be well preserved and, apart from some small regions of the membrane that appeared moth-eaten (as previously described for filoviruses [39]), showed no adverse effects from the fixation process. In thin areas, instances of microtubules, early endosomes, clathrin coated pits, and the cortical actin network could be discerned.

**Figure 6:**
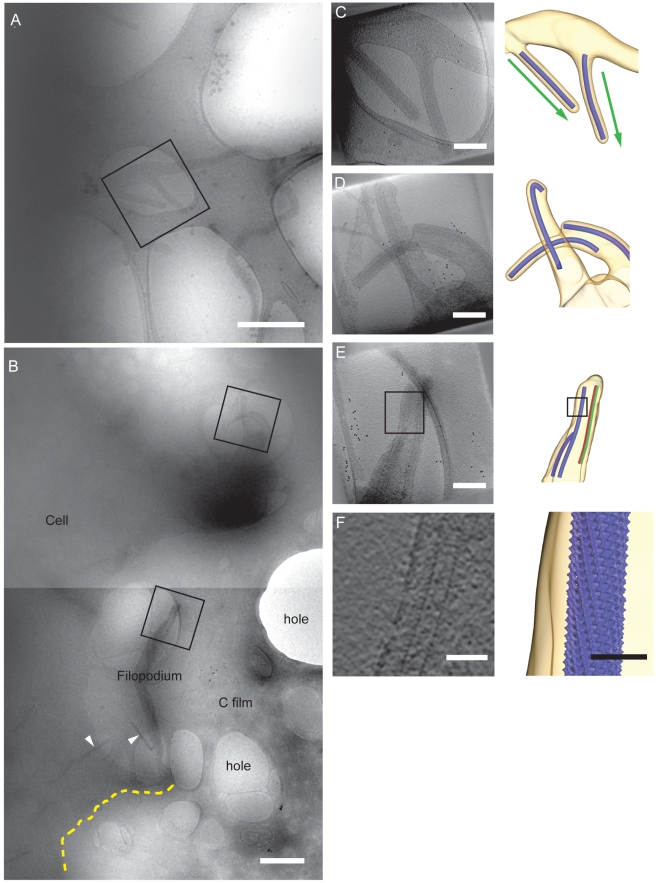
CryoET of MARV budding from infected cells. (A,B) Low magnification images of MARV budding from infected cells. Areas selected as targets for tomography are marked with a black box. The carbon film and examples of holes in the film, the cell, and a filopodium are annotated. NCs in free or budding viruses are marked with arrowheads. Scale bar, 1 µm, electron density black. (C–E) Tomographic reconstructions of targeted regions. Left: projection taken through the reconstructed tomogram. Right: isosurface rendering of the same region. Membrane is shown in yellow and the NC as a blue cylinder. The NC direction, as determined by subtomogram averaging, is indicated by the arrows. An example of membrane-associated (green) and free (red) sides of the NC used for subtomogram averaging is shown in (E). Scale bars, 200 nm. Animations of these tomograms are included as Movies S2, S3, S4. (F) Higher magnification of regions demarcated by boxes in (E) as a slice through the tomogram (left), showing repeating features corresponding to the inner NC helix and as an isosurface representation (right), created by placing the NC reconstruction back into the tomogram. Scale bars, 50 nm.

Many cells displayed a large number of thin, filopodia-like extensions (Figure 6A and 6B), which previous studies have identified as the budding site of MARV [14],[29]. We observed numerous NCs within filopodia and a smaller number of NCs at the plasma membrane of the cell body. Single filopodia frequently contained multiple NCs within their length (Figure 6C, 6D, and 6E). The NCs in the filopodia were almost always associated with the membrane on at least one side, and many partially enveloped NCs were seen extending out from the filopodia, apparently in the process of budding. Repeating features corresponding to the NC helix could be seen in some of the reconstructions (Figure 6F).

**Figure 7:**
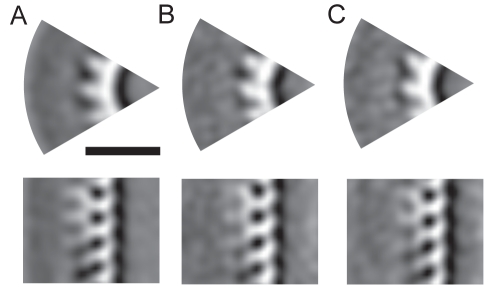
3-D reconstruction of the budding MARV NC. (A) Slice perpendicular to (top) and along (bottom) the NC axis showing a segment of the subtomogram averaging reconstruction of fully enveloped budding MARV NCs (class I). (B) Reconstruction of the membrane associated side of budding NCs that were not fully enveloped on all sides (class II). (C) Corresponding reconstruction of the non-membrane-associated NC (class III).

The NCs were divided into three classes, as follows. NCs which had begun envelopment by the membrane, but which had not yet pinched off from the cell (such as those in Figure 6C) were defined as class I. For NCs that had not yet initiated envelopment, but were associated with the membrane along only one side, the side of the NCs that was associated with the membrane was defined as class II (green in Figure 6E), and the opposite, cytoplasmic side of the NC, was defined as class III (red in figure 6E). We were then able to use subtomogram averaging methods to generate independent 3-D reconstructions of each of these classes, representing different stages in NC envelopment, at resolutions of between 3.6 and 3.8 nm (Figure 7, Materials and Methods). No major differences were seen between the reconstructions up to a resolution of 4 nm. The boomerang-shaped protrusions assigned to VP24 and VP35 were resolved in all cases, as they are in the free virion. These observations suggest that these proteins are already present on the NC prior to association with the viral membrane. Furthermore, no substantial structural changes occur to the NC as a result of membrane association, envelopment, and budding.

### Directionality of NC Transport and Budding

The 3-D reconstructions allowed us to identify the pointed and barbed ends of the NCs within filopodia or budding viruses. Twenty-one NCs that were in the process of budding could have their directionality determined, and all of them were found to be budding with the pointed end of the NC first. This indicates that despite the absence of any dome-like NC tip, factors that induce initial envelopment are specifically associated with the pointed end of the helix: the opposite end to that in rhabdoviruses.

Of the NCs that were found within the filopodia-like extensions, but which had not yet been enveloped by the membrane, it was possible to assign the direction of the NC relative to the filopodia for 26 of them. All of these were found to have the pointed end facing away from the cell, which strongly suggests that there is already directionality inherent in the process by which NCs are transported into the filopodia. Eight viruses were found in early stages of budding where both the direction relative to the filopodia and the direction of budding could be assigned. One of these NCs was oriented with the barbed end facing away from the cell, but was commencing envelopment from the pointed end (Figure S7), suggesting that the directionality of the budding process is independent of the transport process.

## Discussion

### Solving Heterogeneous Structures in situ

The inherent morphological variability of *Mononegavirales* particles has hampered development of a structural understanding of the viruses and their assembly pathways. The bullet-shaped rhabdoviruses appear to be the most structurally regular family within the *Mononegavirales*, and a recent single-particle cryoEM study of VSV [5] provided the first detailed structural view of a member of the order. This study revealed an intimate and regular interaction between the NC with its dome-shaped tip and the matrix layer, providing a potential mechanism for driving assembly and initiation of virus budding. Such single-particle reconstruction methods are not applicable to the other *Mononegavirales* families, such as the filoviruses and the paramyxoviruses, which are much more heterogeneously shaped. Here we have used cryoET to generate low-resolution 3-D structural information of irregularly shaped viruses and have combined it with subtomogram averaging methods to derive higher resolution structures of repeating features. Such methods have a unique advantage in being applicable both to released virions, and potentially also to viruses assembling within infected cells.

Previously, the structures of purified or reconstituted NCs from *Mononegavirales* including MeV and SeV have been solved directly using established image processing methods for the generation of 3-D reconstructions from 2-D electron micrographs of helical objects [6],[7]. These methods are not directly applicable to solve the NC structure from within intact virions because the surrounding matrix layer of the virion is superimposed onto the image of the NC, and this, combined with inherent flexibility in the helix structure, prevents accurate measurement of the helical symmetry parameters. Here we instead used a reference-free subtomogram averaging method to resolve the structure of the NC from within intact virions. This approach allowed the membrane and matrix to be excluded from the analysis, made no prior assumptions about helical parameters, and defined the handedness of the reconstruction. It allowed us to measure helical symmetry parameters accurately, which could then be used to apply established image processing techniques to process a larger dataset and generate a higher resolution reconstruction. Sub-tomogram averaging therefore provides an effective, reference-free method for determining helical parameters and handedness for subsequent 2-D reconstruction in situ that will be applicable to other samples.

The same cryoET and subtomogram averaging techniques that were applied to the free virion were also applied to infected human cells to study viral budding. In this way we were able to generate separate 3-D reconstructions of three different stages of budding in situ. These reconstructions represent uniquely detailed 3-D reconstructions of biological objects within intact cells. CryoET in combination with subtomogram averaging therefore provides a powerful method to derive detailed 3-D structural information for different intermediate steps in biological pathways within intact cells.

### The Structural Organization of Virions

CryoEM and cryoET of MARV virions showed the characteristic range of morphologies and dimensions that have been previously described. The preservation of 3-D structure in the absence of staining permitted the radial density distribution of the particles to be defined, and compared to a radial distribution of proteins obtained using IEM. VP24 and VP35 epitopes were located in the NC region immediately proximal to NP, assigning VP24 as a component of the NC rather as a minor matrix protein as previously suggested [20]. Though unexpected, these data support previous results that Ebola virus VP24 plays a role in formation of a functional NC [40]. It is interesting that VP24, although located at a similar radius as VP35, is released before VP35 upon detergent treatment, suggesting that its interaction with NP [19] is less stable than binding of NP to VP35.

The 3-D reconstructions of MARV NC within intact virions show that MARV possesses a left-handed helical NC, as seen for other members of the order (only RSV has been suggested to have a right-handed NC, but the hand has not yet been experimentally determined [8]). The well-defined inner helix is formed from density lobes that show a clear inclination to the helix axis and that are located at the same radius as the NP epitope localized by our IEM analysis. VP24, VP35, and the C-terminus of NP form a boomerang-shaped protrusion that extends outwards from the inner helix. The VSV NP helix can be directly fitted as a rigid body into the inner helix, demonstrating the presence of a strong architectural homology between the NP helices of the filoviruses and the rhabdoviruses.

A minimal construct NP(1–390) corresponding to the core-conserved region of NP assembled to form a helix with the same hand, pitch, diameter, and inclination as the inner helix of the NC within the virion, showing that the assembly determinants that define the helical structure are contained within the core-conserved domains of NP. In contrast, full-length NP assembled to form loose coils, suggesting that within the virion the disordered C-terminal region may be involved in interaction with other viral proteins, in the absence of which it disrupts NC assembly. A similar effect has been observed previously for MeV and EBOV [7],[41].

We found that although the VP40 layer does contain local ordering, the lattice is not arranged in a defined manner relative to the helical NC. This contrasts with the situation in rhabdoviruses, where there is a fixed and close interaction between NC and matrix layers. Filamentous VLPs that are produced by the expression of VP40 and GP in the absence of an NC also contain a locally regular VP40 lattice. However, this lattice shows differences from that in NC-containing viruses. Together these data imply that formation of a flexible interaction between VP40 and NC induces conformation change in the VP40 lattice.

### The Packing of the Virus Genome

The NP-RNA complex acts as the template for genome replication, and there is therefore an expected relationship between RNA binding and replication. Broadly, members of *Mononegavirales* may be divided into two groups based on genome replication mechanisms. Paramyxoviruses like SeV and MeV have a bipartite replication promoter [42],[43], carry out mRNA editing during transcription of the phosphoprotein gene, and have a total number of nucleotides in the genome that is a multiple of six. Intriguingly, for paramyxoviruses, the number of nucleotides bound to each NP monomer is also divisible by six (the “rule of six”) [44],[45]. On the other hand, rhabdoviruses and pneumoviruses have a monopartite replication promoter [46],[47], do not carry out mRNA editing, and their genome lengths are not multiples of any specific non-unit integer. The number of RNA bases bound per NP monomer range from seven in RSV to nine in VSV and RABV [8],[9],[36].

Here we show that filoviruses package six RNA bases per NP, suggesting that the mechanism of RNA synthesis in filoviruses may be similar to that in *paramyxovirinae* (SeV and MeV). This suggestion is consistent with the observation that the L protein sequences of filoviruses are more similar to those from *paramyxovirinae* than to either RSV or rhabdoviruses. Furthermore, as in SeV and MeV, EBOV undertakes mRNA editing [48], and the replication promoter is bipartite [49]. In EBOV, total genome lengths are not multiples of six, but the length of the spacer region between the two promoter elements needs to be divisible by six for efficient EBOV replication. In *paramyxovirinae* the two promoter elements are located in close proximity to one another on the same face of the helix and it has been postulated for SeV that this allows the viral polymerase to interact simultaneously with both elements [50]. The two promoters and the region between them in MARV cover 75 bases. Here we show that each turn of the MARV NC contains about 180 bases; therefore, the promoter region covers almost half a turn of the first helix. It is possible that during genome replication initiation in the cell, the NC helix adopts a different conformation to bring the two promoter elements closer in space.

### The Mechanism of Virus Assembly

By solving the NC structure in situ at multiple budding stages, we were able to show that both sides of the NC are in a structurally mature form at least as soon as one side of the NC is associated with the membrane. Within the mature NC, alternate NPs within the NC are not equivalent; instead, the boomerang-like protrusion is found between every other NP. How is this non-equivalence introduced? The simplest scenario is that it is introduced at one end of the NC, concomitant with synthesis and assembly of the NC, and propagates along the helix. Alternate NPs in the helix could become non-equivalent through dimerisation of their disordered C-termini of the NP, or dimerisation of 1∶1 stoichiometrically bound associated protein (VP24 or VP35). Alternatively, VP24 or VP35 binding to NP could sterically, or through induction of a conformation change, make the adjacent NP monomer inaccessible for binding.

NP and VP40 are able to assemble independently of one another to form oligomeric assemblies, but must come together during envelopment. Because a precise stoichiometry of interaction, such as that between matrix and NC in VSV, requires perfect alignment of the two assemblies, it can only be achieved by concomitant assembly of the two assemblies, or if one assembles using the other as a template. We found that in MARV association of the NC with VP40 takes place through flexible interactions that induce a rearrangement of VP40, confine the radial position of the VP40 layer and associated membrane, but do not precisely define the lateral position of VP40. Such flexible interactions can also mediate envelopment of a preformed NC by a preformed VP40 lattice through a Velcro-like interaction. This mechanism of interaction may be more robust and would permit significant heterogeneity in the final virion structure, as observed in the filoviruses and in other members of *Mononegavirales*.

The dome-shaped tip of the VSV NC and the tight interaction of the tip with the matrix protein suggest that in VSV interactions between matrix and NC could structurally induce membrane curvature to initiate envelopment of the virus at the tip of the bullet. In MARV, VP40 was seen to coat the inner side of the membrane at the tip of some virions, but we found no dome-like tip structure on either end of the NC and the NC did not extend into the hemispherical tip of the virion. Filoviruses do, however, initiate envelopment at one end of the NC and bud via a protruding intermediate [14]. We therefore asked whether envelopment in filoviruses is initiated specifically from one end of the NC or occurs stochastically at either end.

### The Directionality of Virus Budding and Transport

The directionality of the NC within the budding site is visible in our 3-D reconstructions of individual NCs. We found that the directionality of the NC in all budding sites was the same, indicating that envelopment is indeed initiated specifically at one end of the NC. Unexpectedly, whereas the barbed end of the NC forms the conical tip and buds out first in VSV [5],[51], all MARV NCs were oriented within buds in the opposite direction: with their pointed end outwards.

We found that the barbed tips of filamentous virions were less regular than the pointed tips. Furthermore, the directionality of the NC within virions showed that the bulges of six-shaped virions are predominantly at the rear of the budding direction. These observations are consistent with a previously proposed hypothesis that release of six-shaped and irregular virions can be driven by dynamic membrane invagination prior to complete envelopment of the NC, during late stages of infection [14].

Despite the striking architectural homology of the filovirus NC with that of the rhabdoviruses, together our data show that in contrast to the rhabdoviruses, the filovirus NC has no dome-like NC tip, the NC interacts in a flexible way with the matrix protein, and it buds with the opposite directionality. The mechanism by which filoviruses initiate envelopment is therefore likely to be fundamentally different to that of the rhabdoviruses, but it is likely that at the pointed end of the NC a viral or cellular component is present that can induce structural arrangement of VP40 to form a hemispherical cap. Intriguingly whereas in VSV the 3′ end of the genome is located at the barbed end of the NC, and buds first [5], in RSV the RNA is bound on the opposite side of the NC [8]. If the filoviruses were to assume the same packing mode as RSV, then although the NC buds in the opposite direction to VSV, the packaged RNA would still bud with the 3′ end first. Such a model is attractive: it implies that the first base of the genome to be synthesized is the first to bud, and would allow the absolute directionality of budding to result from the absolute directionality of the RNA.

Surprisingly, there is also a strong (but not absolute) preferential orientation of the NC during transport into filopodia. This directionality is the same as the directionality of budding, suggesting that the same end of the NC is also directing transport into filopodia. In the one case where an NC was oriented within the filopodium in the opposite direction, the direction of budding was still preserved. How MARV NCs are transported into filopodia remains unknown.

### Conclusion

We used cryoET in combination with subtomogram averaging as a powerful method to derive detailed 3-D structural information on filovirus particles, and of the process of virus assembly and budding within intact infected cells. All members of *Mononegavirales* package their RNA genome in helical NCs. They must all transport and recruit the NC to the surface of the cell where it must be enveloped by, and bud through, the plasma membrane to generate infectious particles. The structural data reveal clear architectural homology between filovirus NCs and those of the rhabdoviruses. In contrast, we find that envelopment and budding, a conserved step in the lifecycle, are carried out by quite different mechanisms. We expect the principles of filovirus assembly to be relevant to the other heterogeneously shaped members of *Mononegavirales*.

## Materials and Methods

### Preparation of Virions and Infected Cells

Particles of MARV that were released from infected Vero cells were collected 3 d post-infection, purified by centrifugation through a 20% sucrose cushion, resuspended in PBS, and fixed with PFA prior to release from BSL-4 conditions. For investigation of budding viruses, HUH-7 cells were grown on EM grids, infected with MARV under BSL-4 conditions, and fixed with PFA 22 h post-infection.

### Purification of Recombinant MARV NP and GP-VP40 VLPs

HEK 293 cells were transfected with plasmids encoding either full-length MARV NP or its (1–390) truncation mutant. Cells were lysed 3 d after transfection, and NCs were purified by CsCl gradient centrifugation. GP-VP40 VLPs were made and purified as described previously [38].

### IEM

Virus pellets were fixed, processed for IEM by gelatin embedding (“the Tokuyasu method”), thin-sectioned, and immunolabelled with antibodies against MARV proteins as described elsewhere [33]. EM was performed and immunolabelled radial virion cross-sections were identified. The distances between the centers of viral cross-sections and gold beads were measured for 100–500 gold beads per antibody, and the observed distributions corrected to account for the non-Gaussian nature of this distance measurement.

### CryoEM

For cryoEM studies, vitrified samples were imaged under standard low-dose conditions in a FEI CM120 Biotwin microscope, or for tomography in an FEI TF30 Polara TEM (300 kV) with energy filter. Tomographic tilt ranges were typically from +60° to −60° with a total dose of 6,000–10,000 e^−^/nm^2^.

### Image Processing

Tomograms were reconstructed using the IMOD software suite [52]. Subtomograms were extracted along the length of NCs, and iteratively aligned in six dimensions, taking into account the missing wedge. Subtomogram processing was carried out using scripts derived from the AV3 software package [53] within Matlab. Fourier analysis was carried out using Matlab. Helical reconstruction was carried out using the iterative helical real space reconstruction technique [34].

## Supporting Information

Figure S1 Radial distribution of MARV proteins. Purified MARV particles were fixed and embedded in gelatin. Thawed 60 nm cryosections were immunolabeled using antibodies against MARV proteins NP, VP24, VP35, VP40, or GP followed by protein-A gold. (A) EM micrograph showing thin-sectioned MARV particles, labeled for NP. Boxed area outlines a NP-labeled radial cross-section of a filamentous virus. Scale bar, 100 nm. (B) Panel of NP-labeled radial cross-sections of filamentous viruses, computationally extracted from a stack of EM micrographs. Boxed image highlights the virus cross-section shown in (A). (C–G) Distribution of radial distances of protein-A gold beads from the centre of virus cross-sections for each of the labeled MARV proteins. See Table S1 for further details.
(TIF)

Figure S2 Flexibility of the MARV NC. (A) A central slice through a tomogram of a MARV particle is shown. Blue cones have been placed on positions where the centres of aligned subtomograms project onto a cylindrical surface whose axis is the same as that of the NC helix. The orientation of the cone indicates the output angles from the alignment procedure. The helical nature of the two regions is visible. Scale bar, 100 nm. (B) Slices through the isosurfaces of the reconstructions from each region are shown, and it is clear that the number of subunits per turn varies between the two regions.
(TIF)

Figure S3 Resolution anisotropy in the MARV NC. (A) The Fourier Shell correlation (FSC) curve (solid line) is shown for the MARV NC helical reconstruction presented earlier in Figure 3C–D. The point where the FSC crosses 0.5 is marked (dotted line). (B) FSC at 2.6 nm resolution at different radial distances from the central axis of the virus are plotted (solid line). The radial distance that corresponds to the centre of the NP layer is marked with an arrow.
(TIF)

Figure S4 Core-conserved region of the MARV NP. (A) IUPRED intrinsic disorder prediction score is plotted along the length of the NP sequence (blue). The number on the horizontal axis corresponds to the position along the NP sequence. The C-terminus of the MARV NP is thus predicted to contain large disordered regions, as observed previously for EBOV (B) Windowed conservation scores (proportion of identical residues in a window of length 10) after alignment of EBOV and MARV NP sequences (red). The numbers on the horizontal axis correspond to position in the MARV NP sequence. This analysis highlights a region near the N-terminus of the MARV NP that is conserved with the EBOV NP. This region also shares weak homology with the core conserved part of NPs from other members of *Mononegavirales*.
(TIF)

Figure S5 Directionality and structure of MARV tips. (A) CryoEM image of a long MARV particle with length greater than twice the length of an average virion. The particle is captured in its entirety by the field-of-view of the micrograph. Arrows placed on the virion are directed towards the pointed end of the NC. The positions of the arrows represent the centres of the extracted boxes. Green indicates high cross-correlation of alignment and yellow indicates low cross-correlation. The directionality of the NC does not change along the length of the virus. Scale bar, 100 nm. (B) Two-dimensional average of all “barbed” tips of the MARV. (C) Average of a random subset of “pointed” MARV tips, equal in size to the “barbed” tips dataset. (D) Same as (C), but with a different randomized subset. Scale bar, 50 nm. (E) One single extracted “barbed” tip. (F) One single extracted “pointed” tip.
(TIF)

Figure S6 Arrangement of the VP40 layer. (A) Slice through tomograms of native MARV (left) and GP-VP40 VLPs (right). Scale bar, 100 nm. Some slices show disordered protein densities in the center of viral NCs or VLPs. (B) Corresponding radial density profiles through cross-sections of the particles, calculated by subtomogram averaging (Text S1). The membrane (M), the outer VP40 layer (O), the inner VP40 layer (I), and the NC layer (N) are indicated. Protein density is white. Scale bar, 10 nm. (C) Corresponding 1-D plots of the radial density for the virion (red) and the GP-VP40 VLP (blue). (D) Averaged power spectrum of an unwrapped, flattened section through the outer VP40 layer for the virion (left) and the GP-VP40 VLP (right). Filament axis is horizontal. Peaks indicate the presence of repeating features with dimensions annotated. See Text S1 for details. (E) Corresponding power spectrum of a flattened section from the inner VP40 layer. For the virions (left), (D–E) show the presence of local regularity within both inner and outer VP40 layers. Features repeat along the filament axis approximately every ∼5.2 nm and ∼6.6 nm in the outer layer and every ∼6.6 nm in the inner layer. The inner layer also shows ordering around the axis of the filament in the inner layer with features repeating approximately every 9.3°. The GP-VP40 VLPs (right) show inner and outer layers of VP40 density with features repeating along the filament axis with a spacing of ∼4.2 nm, ∼5.2 nm, and ∼6.6 nm in the outer layer and ∼6.6 nm in the inner layer. In contrast to the virion, there is no ordering around the filament axis in the inner layer. (F) Power spectrum of a flattened section from the NC layer. The peaks seen correspond to those expected from the helical symmetry of the NC.
(TIF)

Figure S7 Example of MARV NC oriented within the filopdium with opposite directionality. A slice through the tomogram showing an NC budding out of the filopodium in the direction indicated by the arrow. The filopodium and the location of the main cell is marked in the figure and therefore the NC was originally oriented within the filopodium in the opposite direction to all other NCs that were measured. Scale bar, 100 nm.
(TIF)

Table S1 Radial distribution of MARV proteins from IEM. Thawed 60 nm cryosections were immunolabeled with antibodies against MARV proteins NP, VP24, VP35, VP40, or GP followed by protein-A gold. Digital images of labeled sections were recorded in the EM, radial cross-sections of labeled viruses were computationally extracted from the images, and the distances of protein-A gold beads from the center of radial virus cross-section were measured. The data were corrected to account for the non-Gaussian nature of this distance measurement (SEM, standard error of the mean). See Figure 2B, Figure S1, and Text S1 for further details.
(DOC)

Text S1 Supporting materials and methods. Additional details about the materials and methods used in this study along with supporting references.
(DOC)

Movie S1 Fitting of VSV NP into the MARV NC density. The fit of four copies of the VSV NP from a pseudo-atomic model (PDBID 2WYY) into the MARV NC reconstruction is shown in different views. The directionality of the MARV NC is also highlighted. See Text S1 for more details.
(AVI)

Movie S2 CryoET of budding MARV from filopodia of infected mammalian cells. Animation through sequential z-slices of the tomogram presented in Figure 6D. Colored overlay shows a 3-D surface model of membranes (yellow) and representations of the viral NCs (blue).
(AVI)

Movie S3 Tomogram of budding nucleocapsids, shown in Figure 6C. See Movie S2 legend.
(AVI)

Movie S4 Tomogram of budding nucleocapsids, shown in Figure 6E. See Movie S2 legend.
(AVI)

## Abbreviations

2-D: 2-dimensional
3-D: 3-dimensional
BSL-4: biosafety level 4
cryoEM: cryo-electron microscopy
cryoET: cryo-electron tomography
EBOV: Ebola virus
EM: electron microscopy
GP: glycoprotein
IEM: immuno-electron microscopy
MARV: Marburg virus
MeV: Measles virus
NC: nucleocapsid
NP: nucleoprotein
PFA: paraformaldehyde
RABV: rabies virus
RSV: respiratory syncytial virus
SeV: Sendai virus
VSV: vesicular stomatitis virus
