# Supplementary figures and images for: Cryo-Electron Tomography of Marburg Virus Particles and Their Morphogenesis within Infected Cells
Source: PLoS Biol. 2011 Nov 15;9(11):e1001196. doi: 10.1371/journal.pbio.1001196 (PMC3217011; doi:10.1371/journal.pbio.1001196)

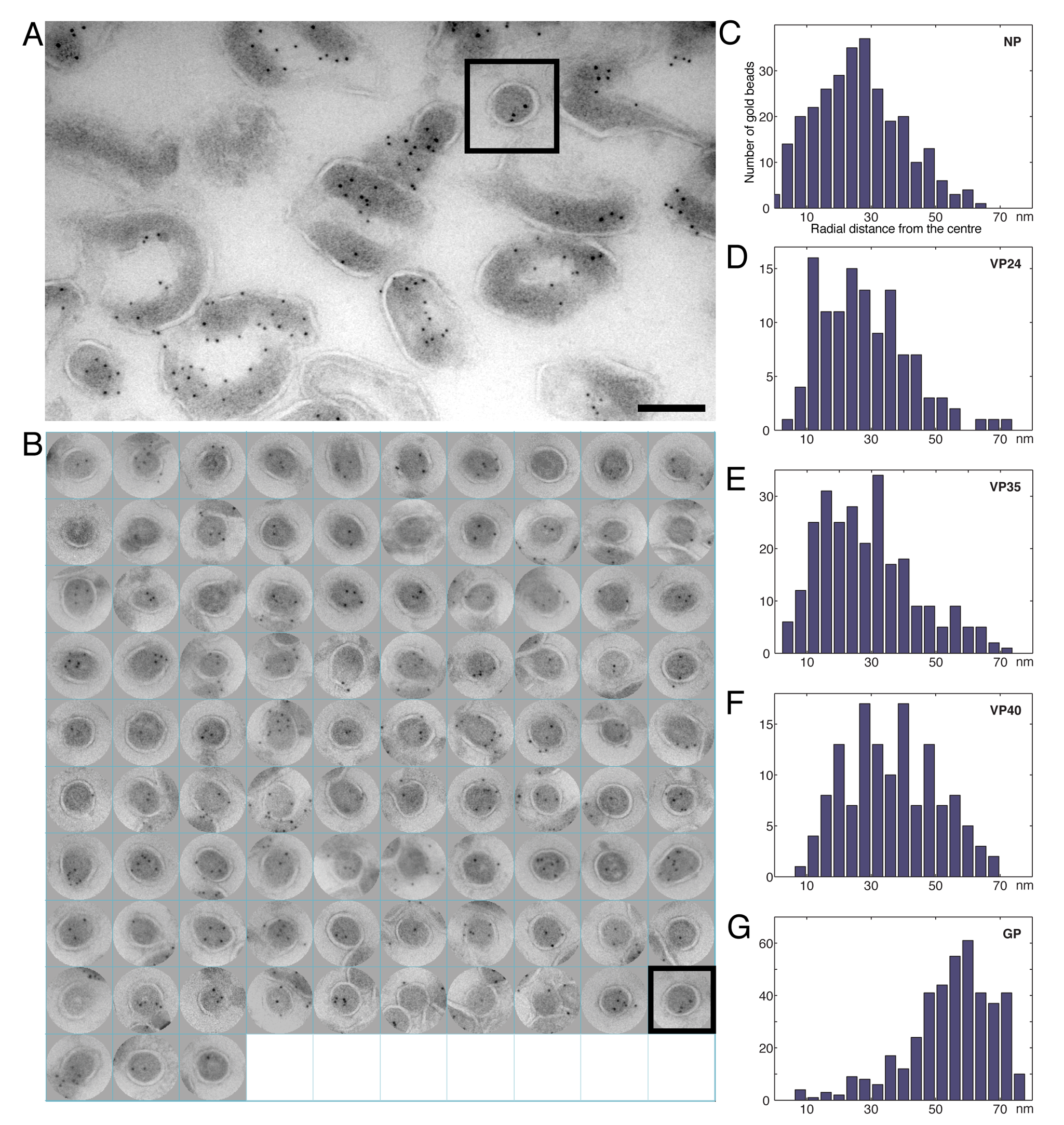

Supplement: Figure S1 — Radial distribution of MARV proteins. Purified MARV particles were fixed and embedded in gelatin. Thawed 60 nm cryosections were immunolabeled using antibodies against MARV proteins NP, VP24, VP35, VP40, or GP followed by protein-A gold. (A) EM micrograph showing thin-sectioned MARV particles, labeled for NP. Boxed area outlines a NP-labeled radial cross-section of a filamentous virus. Scale bar, 100 nm. (B) Panel of NP-labeled radial cross-sections of filamentous viruses, computationally extracted from a stack of EM micrographs. Boxed image highlights the virus cross-section shown in (A). (C–G) Distribution of radial distances of protein-A gold beads from the centre of virus cross-sections for each of the labeled MARV proteins. See Table S1 for further details. (TIF) [file pbio.1001196.s001.tif]

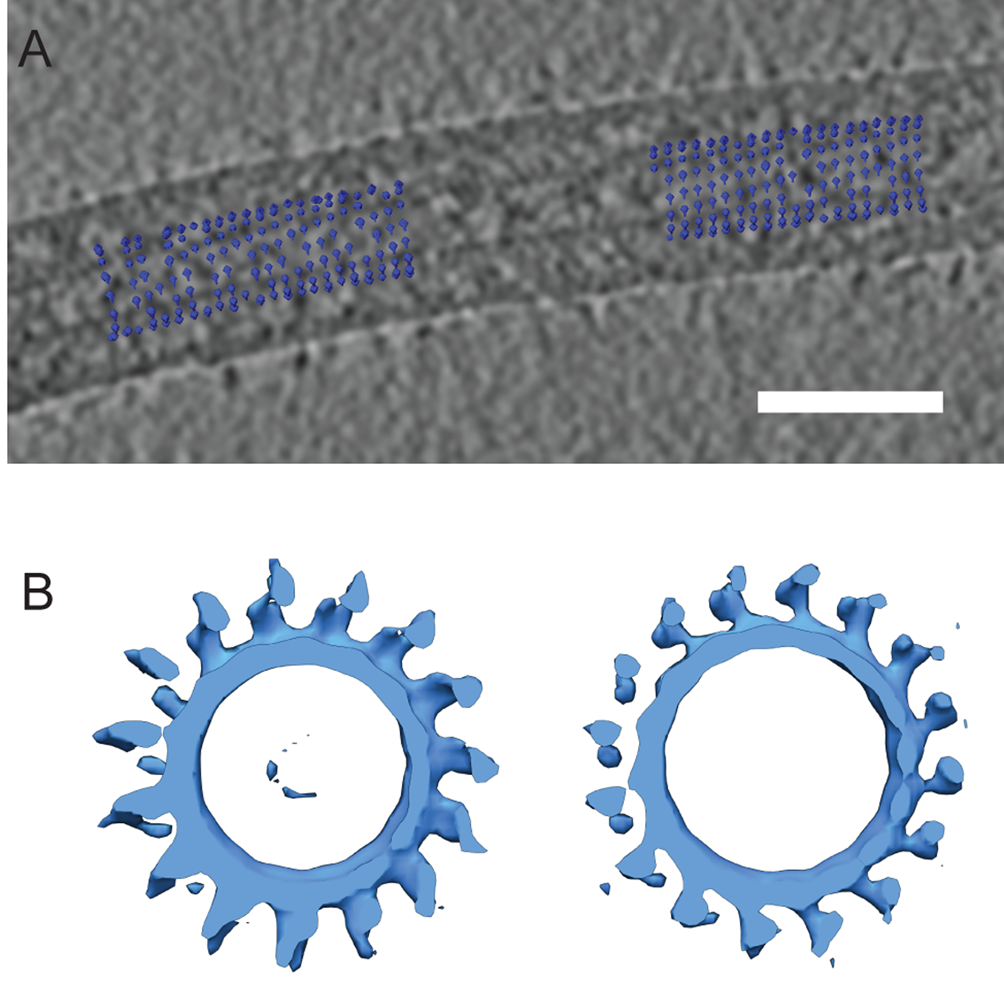

Supplement: Figure S2 — Flexibility of the MARV NC. (A) A central slice through a tomogram of a MARV particle is shown. Blue cones have been placed on positions where the centres of aligned subtomograms project onto a cylindrical surface whose axis is the same as that of the NC helix. The orientation of the cone indicates the output angles from the alignment procedure. The helical nature of the two regions is visible. Scale bar, 100 nm. (B) Slices through the isosurfaces of the reconstructions from each region are shown, and it is clear that the number of subunits per turn varies between the two regions. (TIF) [file pbio.1001196.s002.tif]

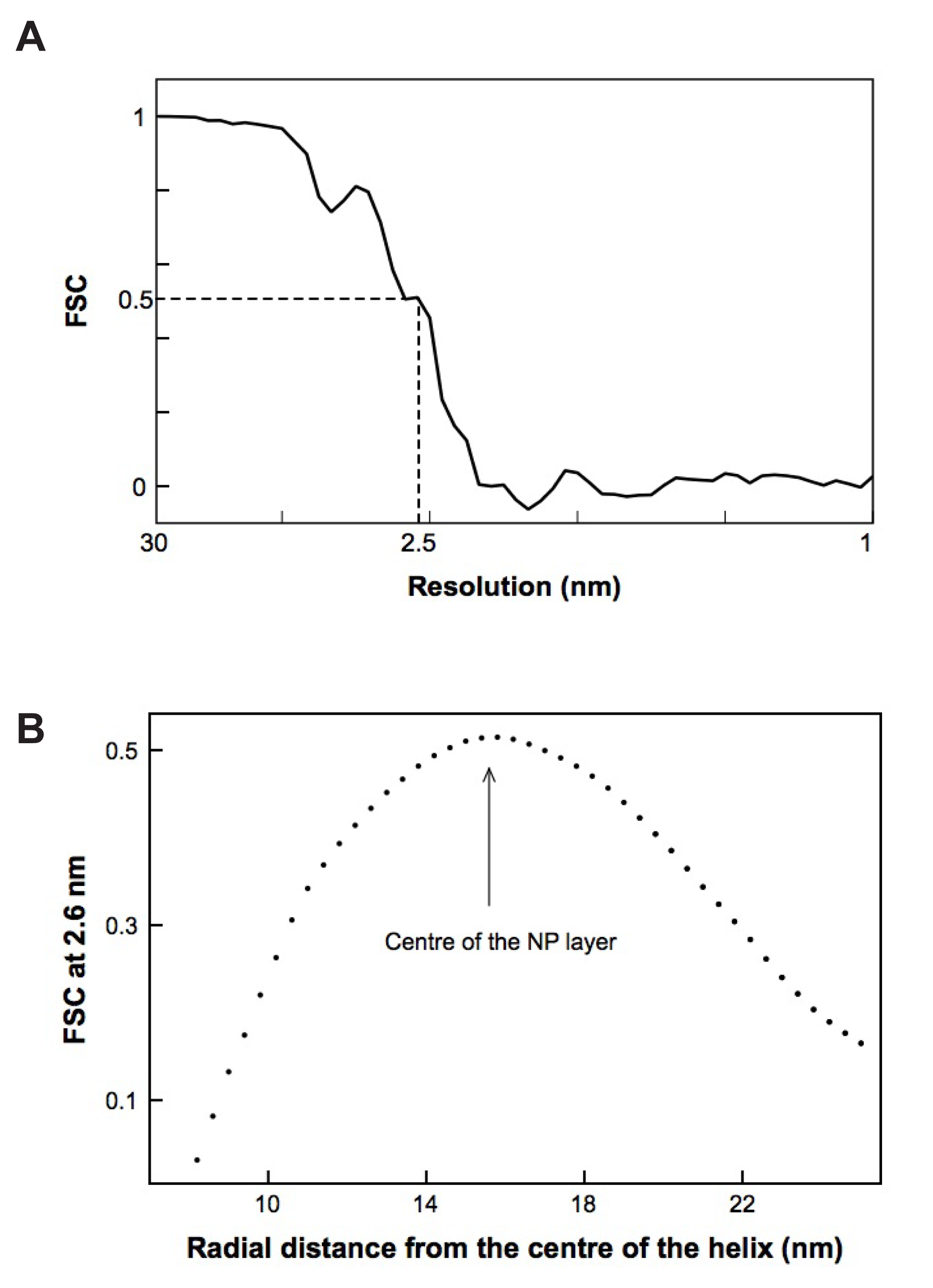

Supplement: Figure S3 — Resolution anisotropy in the MARV NC. (A) The Fourier Shell correlation (FSC) curve (solid line) is shown for the MARV NC helical reconstruction presented earlier in Figure 3C–D. The point where the FSC crosses 0.5 is marked (dotted line). (B) FSC at 2.6 nm resolution at different radial distances from the central axis of the virus are plotted (solid line). The radial distance that corresponds to the centre of the NP layer is marked with an arrow. (TIF) [file pbio.1001196.s003.tif]

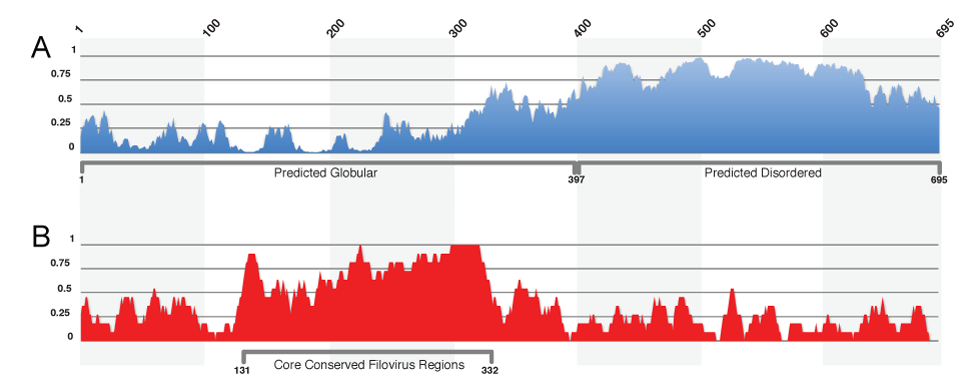

Supplement: Figure S4 — Core-conserved region of the MARV NP. (A) IUPRED intrinsic disorder prediction score is plotted along the length of the NP sequence (blue). The number on the horizontal axis corresponds to the position along the NP sequence. The C-terminus of the MARV NP is thus predicted to contain large disordered regions, as observed previously for EBOV (B) Windowed conservation scores (proportion of identical residues in a window of length 10) after alignment of EBOV and MARV NP sequences (red). The numbers on the horizontal axis correspond to position in the MARV NP sequence. This analysis highlights a region near the N-terminus of the MARV NP that is conserved with the EBOV NP. This region also shares weak homology with the core conserved part of NPs from other members of Mononegavirales. (TIF) [file pbio.1001196.s004.tif]

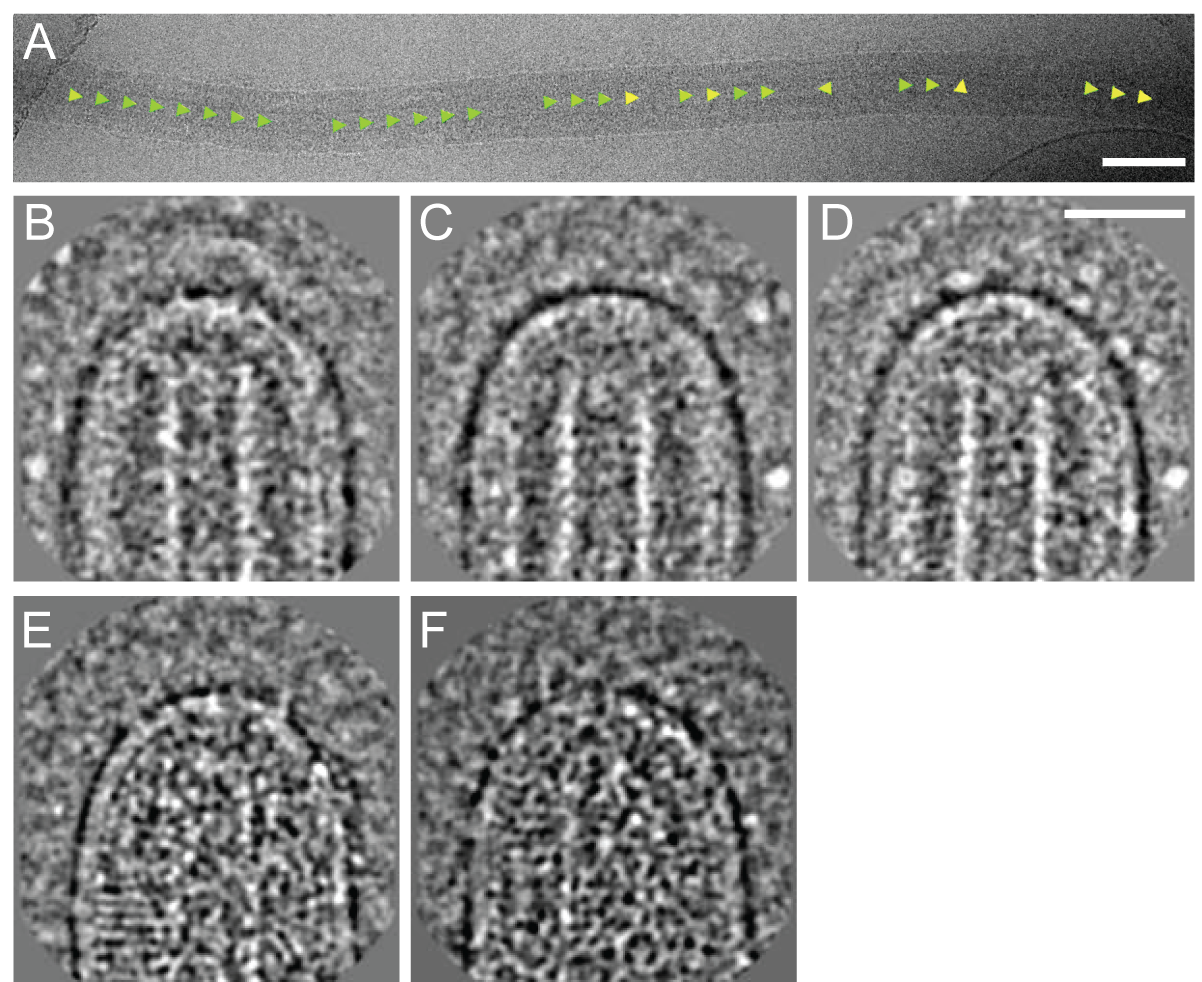

Supplement: Figure S5 — Directionality and structure of MARV tips. (A) CryoEM image of a long MARV particle with length greater than twice the length of an average virion. The particle is captured in its entirety by the field-of-view of the micrograph. Arrows placed on the virion are directed towards the pointed end of the NC. The positions of the arrows represent the centres of the extracted boxes. Green indicates high cross-correlation of alignment and yellow indicates low cross-correlation. The directionality of the NC does not change along the length of the virus. Scale bar, 100 nm. (B) Two-dimensional average of all “barbed” tips of the MARV. (C) Average of a random subset of “pointed” MARV tips, equal in size to the “barbed” tips dataset. (D) Same as (C), but with a different randomized subset. Scale bar, 50 nm. (E) One single extracted “barbed” tip. (F) One single extracted “pointed” tip. (TIF) [file pbio.1001196.s005.tif]

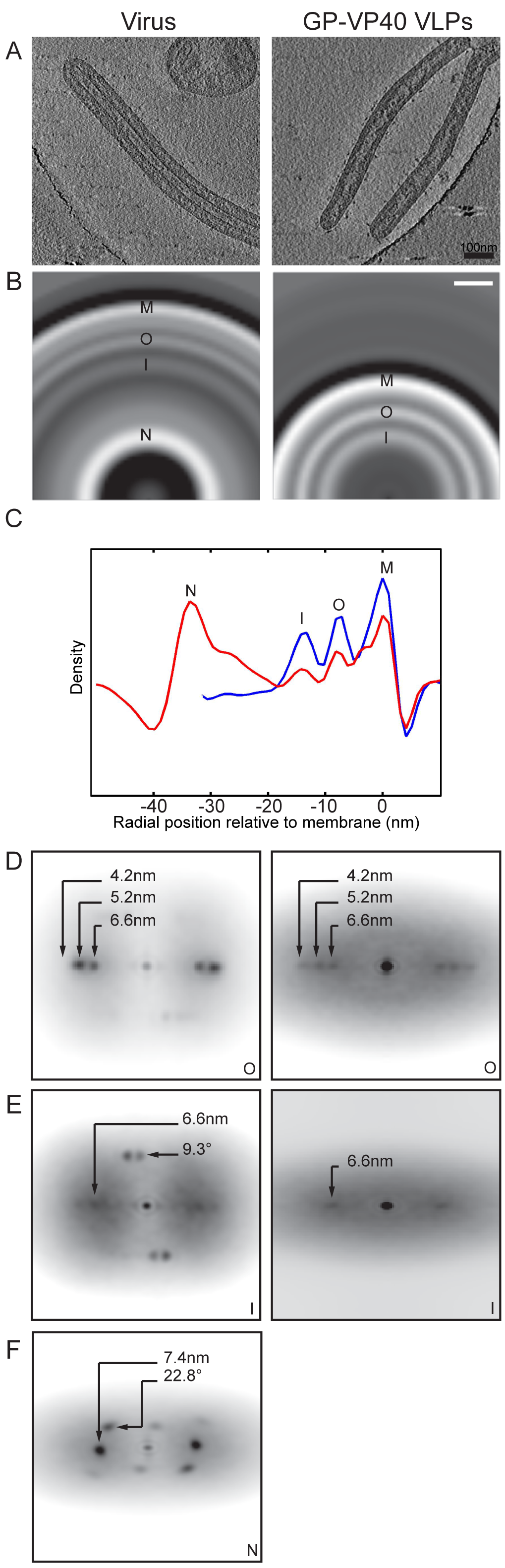

Supplement: Figure S6 — Arrangement of the VP40 layer. (A) Slice through tomograms of native MARV (left) and GP-VP40 VLPs (right). Scale bar, 100 nm. Some slices show disordered protein densities in the center of viral NCs or VLPs. (B) Corresponding radial density profiles through cross-sections of the particles, calculated by subtomogram averaging (Text S1). The membrane (M), the outer VP40 layer (O), the inner VP40 layer (I), and the NC layer (N) are indicated. Protein density is white. Scale bar, 10 nm. (C) Corresponding 1-D plots of the radial density for the virion (red) and the GP-VP40 VLP (blue). (D) Averaged power spectrum of an unwrapped, flattened section through the outer VP40 layer for the virion (left) and the GP-VP40 VLP (right). Filament axis is horizontal. Peaks indicate the presence of repeating features with dimensions annotated. See Text S1 for details. (E) Corresponding power spectrum of a flattened section from the inner VP40 layer. For the virions (left), (D–E) show the presence of local regularity within both inner and outer VP40 layers. Features repeat along the filament axis approximately every ∼5.2 nm and ∼6.6 nm in the outer layer and every ∼6.6 nm in the inner layer. The inner layer also shows ordering around the axis of the filament in the inner layer with features repeating approximately every 9.3°. The GP-VP40 VLPs (right) show inner and outer layers of VP40 density with features repeating along the filament axis with a spacing of ∼4.2 nm, ∼5.2 nm, and ∼6.6 nm in the outer layer and ∼6.6 nm in the inner layer. In contrast to the virion, there is no ordering around the filament axis in the inner layer. (F) Power spectrum of a flattened section from the NC layer. The peaks seen correspond to those expected from the helical symmetry of the NC. (TIF) [file pbio.1001196.s006.tif]

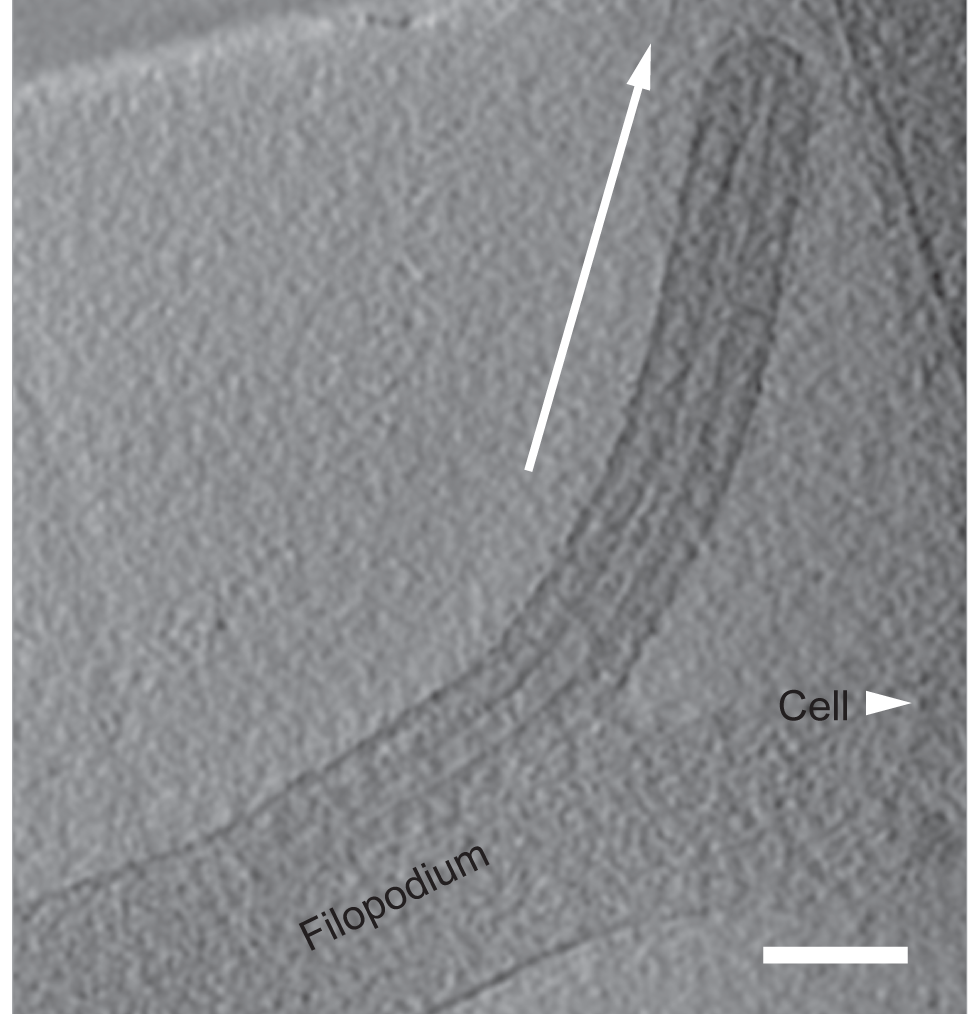

Supplement: Figure S7 — Example of MARV NC oriented within the filopdium with opposite directionality. A slice through the tomogram showing an NC budding out of the filopodium in the direction indicated by the arrow. The filopodium and the location of the main cell is marked in the figure and therefore the NC was originally oriented within the filopodium in the opposite direction to all other NCs that were measured. Scale bar, 100 nm. (TIF) [file pbio.1001196.s007.tif]
