# Supplementary material for: Cryo-Electron Tomography of Marburg Virus Particles and Their Morphogenesis within Infected Cells
Source: PLoS Biol. 2011 Nov 15;9(11):e1001196. doi: 10.1371/journal.pbio.1001196 (PMC3217011; doi:10.1371/journal.pbio.1001196)
Supplement: Table S1 — Radial distribution of MARV proteins from IEM. Thawed 60 nm cryosections were immunolabeled with antibodies against MARV proteins NP, VP24, VP35, VP40, or GP followed by protein-A gold. Digital images of labeled sections were recorded in the EM, radial cross-sections of labeled viruses were computationally extracted from the images, and the distances of protein-A gold beads from the center of radial virus cross-section were measured. The data were corrected to account for the non-Gaussian nature of this distance measurement (SEM, standard error of the mean). See Figure 2B, Figure S1, and Text S1 for further details. (DOC) [file pbio.1001196.s008.doc]

**Table S1. Radial distribution of MARV proteins from IEM.**

| **Antibody Labeling** | **Number of virus profiles** | **Number of gold beads** | **Average radial distance** | | **SEM** |
| --- | --- | --- | --- | --- | --- |
|  |  |  | **before correction (nm)** | **after correction (nm)** | **(nm)** |
| **NP** | 93 | 288 | 25.98 | 18.63 | 0.79 |
| **VP24** | 100 | 118 | 28.12 | 21.57 | 1.25 |
| **VP35** | 153 | 262 | 28.61 | 22.28 | 0.91 |
| **VP40** | 89 | 135 | 36.47 | 32.13 | 1.22 |
| **GP** | 88 | 416 | 54.80 | 52.00 | 0.66 |
