# Supplementary material for: Cryo-Electron Tomography of Marburg Virus Particles and Their Morphogenesis within Infected Cells
Source: PLoS Biol. 2011 Nov 15;9(11):e1001196. doi: 10.1371/journal.pbio.1001196 (PMC3217011; doi:10.1371/journal.pbio.1001196)
Supplement: Text S1 — Supporting materials and methods. Additional details about the materials and methods used in this study along with supporting references. (DOC) [file pbio.1001196.s009.doc]

**SUPPORTING TEXT S1**

**Supporting Materials and Methods**

**Preparation of viruses, VLPs and infected cells**

Particles of MARV that were released from infected Vero cells were collected 3 days after infection and purified by centrifugation through a 20% sucrose cushion in a Beckman SW32 rotor at 25,000 rpm for 2 h, the resulting pellets were resuspended in PBS and then fixed with 4% PFA for 48 h using standard procedures designed to completely inactivate the samples. After fixation, the sample was released from BSL-4 conditions and then centrifuged again in a Beckman TLA45 rotor at 45,000 pm for 30 min. PFA was removed and the pellet was resuspended in 50 – 100 µL of freshly prepared 4% PFA. For investigation of budding viruses, HUH-7 cells were grown on gold holey carbon grids and were then infected with MARV under BSL-4 conditions. At 22 h post infection the samples were fixed in the cell culture dish with 4% PFA for 48 h before being removed from the BSL-4 laboratory. GP-VP40 VLPs were purified from the supernatant of 293 HEK cells transfected with plasmids encoding GP and VP40, and fixed in 4% PFA as described earlier [4,5].

**Purification of recombinant MARV NP**

HEK 293 cells were transfected with plasmids encoding either full length MARV NP or a truncation mutant (consisting of only amino acids 1-390). Cells were lysed three days after transfection by incubating for 30 min at 4°C in TNE (10 mM Tris-HCl, 150 mM NaCl, 1 mM EDTA pH 7.8) buffer containing 1% NP40 and a protease inhibitor cocktail (Roche). Cell lysates were pre-cleared via centrifugation at 20,000 x g, 4°C for 10 min and loaded on top of a discontinuous 20-40% CsCl gradient and centrifuged at 52,000 rpm, 12˚C for 2 h, in a Beckman SW60 rotor. Visible bluish bands containing protein were collected, diluted in TNE buffer and pelleted at 45,000 rpm for 1 h in a Beckman TLA45 rotor. The resulting pellet was resuspended in 30 µL PBS.

**Immunoelectron microscopy (IEM)**

Fixed, purified virus pellets were processed for immunoelectron microscopy as described elsewhere [6,7,8]. Pellets were embedded in 12% gelatin, infiltrated with 2.3 M sucrose in phosphate buffer, mounted onto aluminium pins and flash-frozen in liquid nitrogen. 60 nm thin sections were cut with a Leica EM UC6 microtome, FC6 cryochamber (Leica Microsystems, Wetzlar, Germany) and a diamond knife (Diatome, DiS-Galetzka Weinheim, Germany). Thawed cryosections were immunolabeled with antibodies against MARV proteins VP24, VP35, VP40, GP and NP followed by 5 nm protein-A gold (University of Utrecht, Utrecht, Netherlands). For NP (number of gold beads included in the analysis, n=288), GP (n=416) and VP40 (n=135) monoclonal and for VP24 (n=118) and VP35 (n=262), polyclonal antibodies were used. Thin sections were examined in a systematic random sampling manner [9] on a FEI Morgagni 268 TEM equipped with a 1K side mounted CCD camera (SIS, Muenster, Germany) and digital images (pixel size 0.87 nm) were recorded. Coordinates of the centers of radial virus cross-sections and of protein-A gold beads were identified using the AMIRA EM package [10]. Distance measurements were performed with Matlab.

**Analysis of radial distribution of MARV proteins.**

Radial cross-sections of filamentous MARV particles were identified in a stack of digital images of labeled thin sections for each of the immunolabelings against MARV NP, VP24, VP35, VP40 and GP, and the coordinates of their centres were manually clicked using the AMIRA EM package [10]. Circular regions of radius 79 nm around the virus centers were computationally extracted from the digital images using Matlab (Mathworks). Protein-A gold bead positions were manually clicked using the AMIRA EM package, and their distances from the centers of the masks were measured and displayed (Figure S1 C to G) using Matlab. Radial distance from the helical axis is always a nonnegative number, therefore it cannot be Gaussian distributed. Accurate determination of the mean radial position of viral proteins requires a proper description of distance distribution, which can be obtained by correcting to account for the non-Gaussian nature of this distance measurement (assuming average length of the antibody label is 15.6 nm) based on a mathematical treatment described elsewhere [3]. Final distance values are given in the text and in Table S1. We note that various factors including compression or expansion during sample preparation, or inclusion of elliptical cross-sections could lead to a small overestimate or underestimate of radial positions but would not lead to a change in the relative positions of the proteins. The measured positions of the GP and NP are consistent with the cryo-EM radial density profiles suggests that these effects are not significant.

**Grid preparation and Electron Microscopy**

For cryoEM studies, 3 µl of undiluted sample was pipetted onto a glow discharged, 300-mesh C-Flat grid with 2 µm holes. For tomography, the sample was mixed with a solution of protein-A 10 nm gold in the sample buffer before pipeting onto the grid. The sample was blotted and plunge-frozen in liquid ethane either with a Vitrobot (FEI, Eindhoven) or with a manual plunging device (EMBL Heidelberg) and stored in liquid nitrogen. Infected cells grown on EM grids were blotted from the rear in the manual plunging device in order to minimize damage to the cells. Samples for tomography were transferred at a temperature below -160°C into an FEI TF30 Polara TEM (300kV). Images were collected at a magnification of 27,500 for purified viruses, or 22,500 for infected cells, with an unbinned pixel size of 0.49 or 0.60 nm respectively. Zero loss energy filtered images were collected on a Gatan GIF 2002 post column energy filter and the total dose used was between 6000 – 10000 e^-^/nm^2^. Tilt ranges were typically from +60° to -60°. 2D cryoEM data collection for helical reconstruction was performed on a FEI CM120 Biotwin microscope equipped with a Gatan 626 cryo-holder and a TVIPS 4kx4k CCD camera. In these cases, data was either collected on film at 37,000 magnification or with CCD camera at 33,000 magnification giving an unbinned pixel size of 0.36 nm.

**Subtomogram averaging reconstruction of the NC**

Tomograms were reconstructed using the IMOD software suite [11]. Nucleocapsids that had more than approximately 250 nm of their length in the tomogram were considered to be of sufficient length and were processed as follows. Points along the central axis of the NC in tomograms were picked manually, and a line was spline fitted to these points. Cubic volumes, or subtomograms, with dimensions of 77x77x77 nm^3^ subtomograms were extracted at an interval of 7 nm along the longitudinal axis of the NC helix. Eighteen subtomograms were extracted at each individual point, with the only difference being the angle of rotation about the filament axis of each subtomogram. This scheme was used to minimize the angular search parameters that were required for alignment of the subtomograms. In order to produce an unbiased starting reference, all of the extracted subtomograms were summed together, rotationally averaged about the longitudinal axis of the virus and projected along this same axis. Individual subtomograms were aligned in six dimensions against a missing wedge corrected reference using scripts based on the AV3 package [12]. After alignment, subtomograms were shifted, rotated and then averaged to produce a new reference. This process was iterated until a stable solution was obtained. The helical symmetry parameters, such as the number of subunits per turn and the pitch, could be measured directly from the reference. They could also be obtained through inspection of the lattice map, which shows the final positions and rotations of all subtomograms after alignment.

The directionality of the budding NCs was assessed by inspection of the innermost density of the helix in the final reference that was generated by subtomogram averaging. NCs were only processed further if their helical symmetry could be determined, and if the quality of the final reference from initial subtomogram averaging was considered to be of reasonable quality. NCs were divided into three groups, as explained in the results. From our total dataset of 68 NCs, this gave us 17 NCs that were assigned to class I, and 16 NCs that were assigned to classes II and III. For classes II and III, the region of the NC covering a 120° arc facing towards or away from the membrane, respectively, was included in the average. No substantial differences were observed between NC reconstructions of different classes: only insignificant differences comparable in scale to differences between reconstructions of two halves of the same class were observed. All subtomogram processing was carried out using Matlab (Mathworks) with scripts adapted from the TOM and AV3 software packages [12]. Visualization of volumes was done using Amira (Visage Imaging GmbH).

**Radial density profiles and Fourier analysis**

Subtomograms extracted at the surface of the particles were radially aligned to the membrane of the virion (or the VLP). The rotational average of the aligned subtomograms about the particle centre generated a radial density profile (Figure S6-B). The aligned subtomograms were converted to cylindrical co-ordinates (r,Φ,z) (effectively unwrapping the outer layers of the virion to make them flat). For each value of r, a 2D power spectrum was calculated, and the power spectra for all individual subtomograms were summed to produce an averaged power spectrum for each radial layer. At a given radial layer in a virion, any repeating features will give rise to a peak in the power spectrum (equivalent to the peak in the diffraction pattern of a 2D crystal). The position of the peak in the x axis corresponds to the frequency along the axis, while the position in the y axis corresponds to the frequency around the axis of the virion. Power spectra from the two membrane adjacent VP40 layers in virions and GP-VP40 VLPs is shown are shown in Figure S6.

**2D CryoEM Image Processing**

Electron micrographs were scanned using a ZEISS SCAI scanner at 14 µm pitch, giving a pixel size of 0.4 nm. 80x80 nm^2^ Particle boxes were picked at 90% overlap with the BOXER program from the EMAN [13] suite. CTF correction of the images was carried out using the Bsoft software package [14]. Three-dimensional reconstruction was carried out using the iterative helical real space reconstruction technique [15] in the Spider package [16]. Different starting parameters centered around the parameters extracted from tomography were applied in independent runs. The parameters for helical shift (∆Z) and helical rotation (∆φ) converged to (0.50 nm, 24.33°) and (0.47 nm, 22.81°) respectively for the two major symmetries of the MARV NC. These parameters were used for subsequent refinement.

For the NP(1-390) mutant, from cryoEM CCD images, ~1200 72x72 nm^2^ boxes suitable for image processing were extracted. Reconstruction using the same helical parameters as that of the MARV NC was carried out as described above. The resolution of the reconstruction was approximately 5 nm. Molecular graphics images were produced using the UCSF Chimera package from the Resource for Biocomputing, Visualization, and Informatics at the University of California, San Francisco (supported by NIH P41 RR001081) [17].

To map directionality information of the NC back onto scanned micrographs, alignment of the original data with reprojections of the MARV NC reconstruction was carried out. Arrows were placed on micrographs using the EM package in Amira. Of the 164 tips with unambiguously assigned directionality, 119 were pointed tips and 45 were barbed tips. There were fewer barbed tips because in six-shaped particles, in most cases the barbed end of the NC is within the bulge of the ‘six’. To control for the different dataset sizes, randomized subsets of the pointed tips were considered, these are shown in Figure S4. Additionally, within the set of 164 tips, we identified 9 tips where VP40-like striations were visible. 8 of these were pointed tips and 1 was a barbed tip.

**Fitting of the pseudo-atomic model of the VSV NP structure into the MARV NC density**

The MARV NC density contains multiple proteins. By comparison with the structure of the C-terminal deletion mutant of the MARV NP, we were able to assign the innermost layer of the NC to NP. Following this assignment, we fitted the pseudo-atomic model of the VSV NP derived from the VSV cryoEM structure (PDB ID 2WYY) into the inner-layer of the NC structure. The VSV structure is the only known pseudo-atomic model from native virions [18,19]. This PDB file contains 10 VSV monomers, arranged as a left handed helix, and showing the characteristic inclination of the long-axis of the NP from the helical axis as seen in other *Mononegavirales* NPs. The VSV and MARV NC helices have different diameters, and therefore curvatures. We fitted four adjacent monomers since the displacement due to the different curvatures is smaller over this distance. The PDB structure could only be fitted in one single distinct orientation: other orientations are excluded because they are not consistent with the curvature of the helix, and with the inclination of the monomer to the helical axis. The four adjacent VSV NP monomers from the PDB file (2WYY) were directly fitted in as a single rigid body (without any modification) and optimized using the “fit in map” algorithm within the UCSF Chimera package (v1.3) [17].

**Supporting References:**

1. Dosztányi Z, Csizmok V, Tompa P, Simon I (2005) IUPred: web server for the prediction of intrinsically unstructured regions of proteins based on estimated energy content. Bioinformatics 21: 3433-3434.

2. Noda T, Hagiwara K, Sagara H, Kawaoka Y (2010) Characterization of the Ebola virus nucleoprotein-RNA complex. J Gen Virol 91: 1478-1483.

3. Churchman L, Flyvbjerg H, Spudich J (2006) A non-Gaussian distribution quantifies distances measured with fluorescence localization techniques. Biophys J 90: 668-671.

4. Mittler E, Kolesnikova L, Strecker T, Garten W, Becker S (2007) Role of the transmembrane domain of Marburg virus surface protein GP for the assembly of the viral envelope. J Virol 81.

5. Kolesnikova L, Bamberg S, Berghöfer B, Becker S (2004) The matrix protein of Marburg virus is transported to the plasma membrane along cellular membranes: exploiting the retrograde late endosomal pathway. J Virol 78: 2383-2393.

6. Liou W, Geuze H, Slot J (1996) Improving structural integrity of cryosections for immunogold labeling. Histochem Cell Biol 106: 41-58.

7. Tokuyasu K (1973) A technique for ultracryotomy of cell suspensions and tissues. J Cell Biol 57: 551-565.

8. Peters P (1999) Cryo-Immunogold Electron Microscopy. Current Protocols in Cell Biology. pp. 4.7.1-4.7.12.

9. Lucocq JM, Habermann A, Watt S, Backer JM, Mayhew TM, et al. (2004) A rapid method for assessing the distribution of gold labeling on thin sections. J Histochem Cytochem 52: 991-1000.

10. Pruggnaller S, Mayr M, Frangakis A (2008) A visualization and segmentation toolbox for electron microscopy. J Struct Biol 164: 161-165.

11. Kremer JR, Mastronarde DN, McIntosh JR (1996) Computer visualization of three-dimensional image data using IMOD. J Struct Biol 116: 71-76.

12. Förster F, Medalia O, Zauberman N, Baumeister W, Fass D (2005) Retrovirus envelope protein complex structure in situ studied by cryo-electron tomography. Proc Natl Acad Sci U S A 102: 4729-4734.

13. Ludtke S, Baldwin P, Chiu W (1999) EMAN: semiautomated software for high-resolution single-particle reconstructions. J Struct Biol 128: 82-97.

14. Heymann J, Belnap D (2007) Bsoft: image processing and molecular modeling for electron microscopy. J Struct Biol 157: 3-18.

15. Egelman E (2000) A robust algorithm for the reconstruction of helical filaments using single-particle methods. Ultramicroscopy 85: 225-234.

16. Frank J, Radermacher M, Penczek P, Zhu J, Li Y, et al. (1996) SPIDER and WEB: processing and visualization of images in 3D electron microscopy and related fields. J Struct Biol 116: 190-199.

17. Pettersen EF, Goddard TD, Huang CC, Couch GS, Greenblatt DM, et al. (2004) UCSF Chimera--a visualization system for exploratory research and analysis. J Comput Chem 25: 1605-1612.

18. Green TJ, Zhang X, Wertz GW, Luo M (2006) Structure of the vesicular stomatitis virus nucleoprotein-RNA complex. Science 313: 357-360.

19. Ge P, Tsao J, Schein S, Green TJ, Luo M, et al. (2010) Cryo-EM model of the bullet-shaped vesicular stomatitis virus. Science 327: 689-693.
